# Preparation and Properties of Reversible Emulsion Drilling Fluid Stabilized by Modified Nanocrystalline Cellulose

**DOI:** 10.3390/molecules29061269

**Published:** 2024-03-13

**Authors:** Fei Liu, Yongfei Li, Xuewu Wang, Zhizeng Xia

**Affiliations:** 1 College of Petroleum Engineering, Shandong Institute of Petroleum and Chemical Technology, Dongying 257061, China; 2 Chemistry and Chemical Engineering, Xi’an Shiyou University, Xi’an 710065, China

**Keywords:** reversible emulsion drilling fluid, reversible emulsion, modified nanocrystalline cellulose, reversible emulsifier

## Abstract

Reversible emulsion drilling fluids can concentrate the advantages of water-based drilling fluids and oil-based drilling fluids. Most of the existing reversible emulsion drilling fluid systems are surfactant-based emulsifier systems, which have the disadvantage of poor stability. However, the use of modified nanoparticles as emulsifiers can significantly enhance the stability of reversible emulsion drilling fluids, but ordinary nanoparticles have the disadvantages of high cost and easily causing environmental pollution. In order to solve the shortcomings of the existing reversible emulsion drilling fluid system, the modified nanocrystalline cellulose was considered to be used as an emulsifier to prepare reversible emulsion drilling fluid. After research, the modified nanocrystalline cellulose NWX-3 can be used to prepare reversible emulsions, and on this basis, reversible emulsion drilling fluids can be constructed. Compared with the reversible emulsion drilling fluid stabilized by HRW-DMOB (1.3 vol.% emulsifier), the reversible emulsion drilling fluid stabilized by the emulsifier NWX-3 maintained a good reversible phase performance, filter cake removal, and oily drill cuttings treatment performance with less reuse of emulsifier (0.8 vol.%). In terms of temperature resistance (150 °C) and stability (1000 V < W/O emulsion demulsification voltage), it is significantly better than that of the surfactant system (temperature resistance 120 °C, 600 V < W/O emulsion demulsification voltage < 650 V). The damage of reservoir permeability of different types of drilling fluids was compared by physical simulation, and the damage order of core gas permeability was clarified: water-based drilling fluid > reversible emulsion drilling fluid > oil-based drilling fluid. Furthermore, the NMR states of different types of drilling fluids were compared as working fluids, and the main cause of core permeability damage was the retention of intrusive fluids in the core.

## 1. Introduction

Emulsion is a heterogeneous dispersion system [1] in which one liquid is dispersed in the form of tiny droplets in another that is not miscible with it [2]. Under certain conditions, the emulsion liquid state can be transformed between O/W emulsion (oil-in-water emulsion) and W/O emulsion (water-in-oil emulsion), that is, “response” to external conditions [3], and this phase change in reversible emulsions can be reversed. These external conditions refer to the reversible phase control method, including the single and two joint responses of pH, temperature, salinity, phase volume, light, CO_2_, and the hydrophilic lipophilic emulsifier ratio. The joint response is reported including the pH and temperature co-response, pH and salinity co-response, and temperature and shear co-response. Among these, reversible emulsions with a pH response have attracted research interest in the field of drilling fluids due to the easy control of the reversible phase [4,5,6,7,8,9,10]. The principle is that by changing the pH value of the emulsion (adding acid or alkali), the hydrophilic lipophilic balance value (HLB value) of the emulsifier is adjusted, so that the emulsion can be transformed between the W/O emulsion and the O/W emulsion.

Patel et al. used organic amine surfactants as reversible emulsifiers. The primary amine was modified into a W/O emulsifier by diethoxidation or monoalkylamination to stabilize the W/O emulsion. When phase conversion is required, the emulsifier is transformed into an O/W emulsifier by protonation, and the emulsion is transformed into O/W emulsion. The protonation and deprotonation of emulsifiers are reversible, and the direction of the reaction can be controlled by acid/base to achieve the reversible phase of the emulsion [11]. Li et al. built a pH-sensitive reversible emulsion system based on supramolecules with a pH-response function [12]. Lin et al. proposed that the HLB value of oxidized fatty acids has acid/alkali-response properties and can also be used to stabilize reversible emulsions [13]. Lan et al. reported that Fe_3_O_4_ nanoparticles adsorbed by an oleic acid bilayer, with a carboxyl group outside, can respond to pH changes [14]. E.s. Read et al. applied ionizable polymer-coated polystyrene latex particles as an emulsifier to stabilize Pickering emulsion. The surface of the emulsifier particles was hydrophilic under acidic conditions, which could stabilize an O/W emulsion. It is oleophilic under alkaline conditions and can stabilize the W/O emulsion [15]. Byuji Fujii et al. studied 4-vinylpyridine-modified SiO_2_ nanoparticles with a pH-response function, the principle of which was to regulate the protonation and deprotonation of the N atom of 4-vinylpyridine through the change in pH value [16]. Tu et al. prepared a solid particle, called the Janus particle, which changes its shape and hydrophilic and oleophilic properties with the change in pH value, and thus changes the type of stabilized emulsion [17].

Water-based drilling fluid and oil-based drilling fluid are the two most widely used drilling fluids [18]. Compared with the water-based, oil-based drilling fluid has several advantages, including a strong inhibition performance, good high-temperature resistance, and lubrication. The former can stabilize well walls, prevent collapse, and has a good protection performance for oil and gas reservoirs, especially water-sensitive formations. The latter is suitable for deep wells and high-inclination wells and is conducive to drilling long horizontal wells with regular boreholes in shale formations [19]. Therefore, shale reservoir development has become a hot situation, and oil-based drilling fluids have received more attention than before. However, oil-based drilling fluids also have problems, the most prominent of which is that the wettability of solid surfaces such as drilling cuttings and formations changes from water-wet to oil-wet, resulting in difficulties in mud cake removal during completion [20], low cement bonding strength during cementing [21], formation blockage by emulsion, and the difficult disposal of oily drilling cuttings and drilling waste (2 × 10^6^ tons/year) [22,23,24,25,26]. It seriously restricts its further expansion in application.

To solve this problem, Patel et al. [27] proposed reversible emulsion drilling fluid, which uses chemical methods to control the phase state of the emulsion, so that the drilling fluid can be converted between W/O and O/W emulsions in different stages of drilling and completion. In other words, the drilling stage is a W/O emulsion with the performance of oil-based drilling fluid. It is transformed into an O/W emulsion drilling fluid, which has the properties of water-based drilling fluid. This emulsion phase conversion combines the advantages of oil-based drilling fluid and water-based drilling fluid, fundamentally solves the contradiction between drilling and completion efficiency, and brings new opportunities for the drilling industry, which is considered to be a major leap forward in drilling fluid technology.

Although reversible emulsion drilling fluid has perfect performance characteristics, there are still some problems in the research of reversible emulsion drilling fluid at this stage, which restricts its large-scale popularization and application. Most of the existing reversible emulsion drilling fluid systems are stabilized by surfactant emulsifiers, which have the disadvantage of poor stability. Modified nanoparticles as reversible emulsifier can obviously enhance the stability of reversible emulsion drilling fluid, but ordinary nanoparticles have the disadvantage of high cost and easily causing environmental pollution. Here, modified nanocrystalline cellulose is used as an emulsifier to prepare reversible emulsion drilling fluid to solve the shortcomings of the existing reversible emulsion drilling fluid system and promote the promotion and application of a reversible emulsion drilling fluid system in a wider range.

Cellulose is a renewable natural polymer material with the largest reserves and the largest yield in nature [28,29,30], which is mainly synthesized by natural plants through photosynthesis [31]. It has a supramolecular structure [32], and its spatial aggregation is a system of alternating arrangements of crystalline and amorphous zones, and the arrangement of the crystalline zones is orderly and regular. However, the arrangement of the amorphous regions is relatively loose [33]. The amorphous zone has a high degree of chemical reaction accessibility, and nanocrystalline cellulose can be obtained by removing the amorphous zone in cellulose by physical and chemical means while retaining the crystalline zone [34]. Nanocrystalline cellulose is derived from abundant renewable resources, has biodegradable properties, and has low environmental, health, and safety risks. Nanocrystalline cellulose not only retains the characteristics of cellulose, but also possesses the unique properties caused by the scale effect of nanomaterials. In terms of nanocrystalline cellulose as an emulsifier to stabilize emulsions, S.A. Kedzior et al. proposed that nanocrystalline cellulose can be used as an emulsifier to achieve adjustments to emulsion type, stability, and stimulus-response behavior [35]. Aseem Pandey et al. proposed the introduction of nanocrystalline cellulose to construct an emulsion system for enhanced oil recovery. At present, the application of nanocrystalline cellulose in the field of drilling fluids is mainly reported as a filter loss reducer [36,37,38]. In 2015, Li et al. added nanocrystalline cellulose to bentonite slurry for performance testing and showed a certain performance of reducing filtration loss [39]. Subsequently, scholars in related fields have conducted a lot of research on nanocrystalline cellulose type filter reducers [40,41]. It is clear that nanocrystalline cellulose can exert an excellent high temperature resistance and filtration loss reduction through adsorption, viscosity, and micro and nano sealing and plugging [42].

In this paper, it is proposed to replace the surfactant reversible emulsifier with a modified nanocrystallitic cellulose reversible emulsifier with a wide range of sources, which are renewable, biocompatible, and biodegradable and can improve the temperature resistance of reversible emulsion drilling fluid. Meanwhile, this method can also reduce the high cost and nano pollution problems caused by the use of modified nanocrystallitic silica reversible emulsifier and improve the stability of reversible emulsion drilling fluid on the basis of ensuring environmental protection and low cost.

## 2. Results and Discussion

### 2.1. Preparation of Reversible Emulsion of Modified Nanocrystalline Cellulose

**Figure 1:**
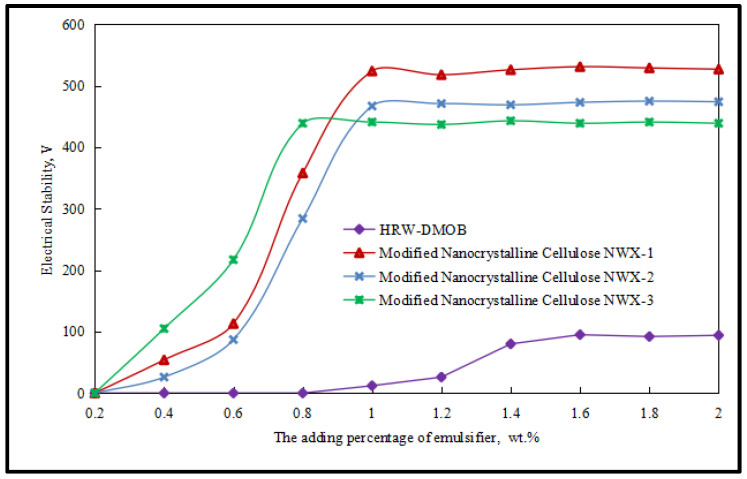
The state of emulsion changes with the dosage of different types of emulsifiers.

Based on previous studies, three kinds of modified nanocrystalline cellulose are selected to prepare stable W/O emulsions. The modified nanocrystalline cellulose NWX-1 was obtained by modifying nanocrystalline cellulose CNC-4 with the primary amine surfactant DUW-3. The modified nanocrystalline cellulose NWX-2 was obtained by modifying nanocrystalline cellulose CNC-4 with the quaternary amine surfactant LHS-5, and the modified nanocrystalline cellulose NWX-3 was obtained by modifying nanocrystalline cellulose CNC-4 with the tertiary amine surfactant LTS-3. Then, it was used as an alternative for the next modified nanocrystalline cellulose reversible emulsifier. The initial emulsions prepared by the three modified nanoparticle reversible emulsifiers were named Initial Emulsion 1, Initial Emulsion 2, and Initial Emulsion 3, respectively, and the acid–base sensitive reversible phase properties of each group of emulsions were tested. Based on previous studies, hydrochloric acid and sodium hydroxide with the same 20.0 wt.% mass fraction were selected as acid phase and alkali phase for the experiment. Firstly, the dosage of the three emulsifiers tested was further optimized. The experimental results are shown in Figure 1.

As shown in Figure 1, a stable O/W emulsion liquid phase ratio can be formed when the dosage of HRW-DMOB is 1.3 wt.% compared with that of the surface-active reversible emulsifier system made by the laboratory. The modified nanocrystalline cellulose NWX-1, NWX-2, and NWX-3 can form a stable W/O emulsion with a small amount (≤1.0 wt.%). The reduction in the amount of emulsifier can not only reduce the cost, but also reduce the environmental pollution caused by the emulsifier. At the same time, the stable initial emulsion of the reversible emulsifier system HRW-DMOB can reach the basically stable value of demulsification voltage of less than 100 V. The demulsification voltages (>400 V) of the modified nanocrystallitic cellulose NWX-1 stable Initial Emulsion 1, modified nanocrystallitic cellulose NWX-2 stable Initial Emulsion 2, and modified nanocrystallitic cellulose NWX-3 stable Initial Emulsion 3 were significantly higher than those of the surfactant system HRW-DMOB-stabilized W/O emulsions when the demulsification voltage was basically stable, indicate that the stability of the W/O emulsions stabilized by modified nanocrystallitic cellulose is significantly higher than that of the W/O emulsions stabilized by HRW-DMOB. In conclusion, on the one hand, the use of modified nanocrystallized cellulose to prepare reversible emulsions can reduce the cost and pollution with less emulsifier, on the other hand, the stability of the initial O/W emulsion prepared by modified nanocrystallized cellulose under the same conditions is significantly higher than that of the initial O/W emulsion prepared by the surfactant system HRW-DMOB. The addition amounts of emulsifiers for preparing reversible emulsion in the next step were determined as follows: the addition amount of the modified nanocrystalline cellulose NWX-1 was 1.0 wt.%, and the addition amount of the modified nanocrystalline cellulose NWX-3 was 0.8 wt.%.

### 2.2. The Phase Transfer Performance of Different Types of Reversible Emulsions

**Figure 2:**
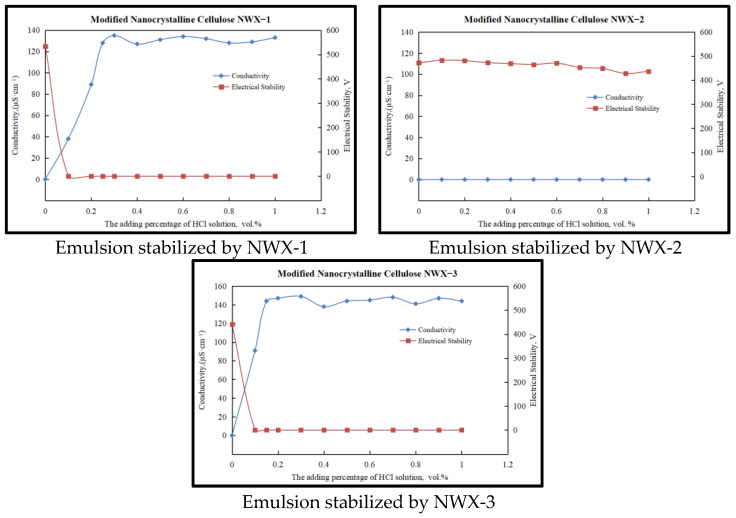
The change in characteristics during the acid-treated phase inversion process of reversible invert emulsion stabilized by different types of emulsifier.

The key to reversible emulsions is to achieve a reversible phase, so it is important to study the phase performance of emulsions formed by different types of emulsifiers. First, the initial Emulsion 1 (1.0 wt.% NWX-1), Emulsion 2 (1.0 wt.% NWX-2), and Emulsion 3 (0.8 wt.% NWX-3) were subjected to acid-response phase conversion (20.0 wt.% hydrochloric acid) experiments, respectively, and the results are shown in Figure 2.

It can be seen that the modified nanocrystalline cellulose NWX-1 and NWX-3 emulsion acids have a good response to phase transfer. However, the NWX-2 emulsion could not complete the acid-responsive phase conversion until the hydrochloric acid addition reached 1.0 vol.%. When the hydrochloric acid dosage reached 1.0 vol.%, the pH value of the emulsion system was as low as 2.9, and the acidity was already strong. At this time, the non-ionic amine group on the surface of the modified nanocrystalline cellulose has been basically converted into ionic amine, and if the emulsion is not phase-transformed under the pH value, it means that the emulsion cannot achieve an acid-responsive phase transformation. Therefore, NWX-1 and NWX-3 were selected as emulsifiers for the next step of alkali-response phase transfer experiment.

**Figure 3:**
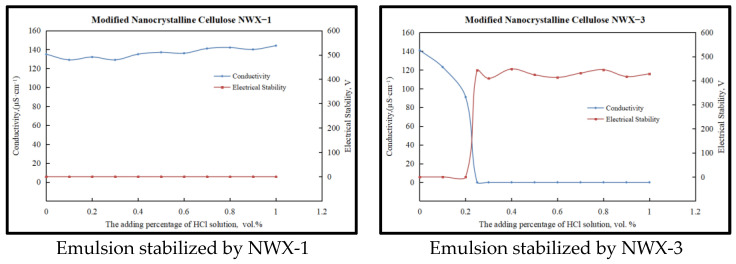
The change in characteristics during the alkali-treated phase inversion process of reversible invert emulsion stabilized by different types of emulsifier.

According to the above experimental results, alkali-response phase conversion experiments (20 wt.% sodium hydroxide solution) were carried out for an NWX-1 stabilized O/W emulsion (0.25 vol.% hydrochloric acid) and an NWX-3 stabilized O/W emulsion (0.15 vol.% hydrochloric acid), and the experimental results are shown in Figure 3.

**Figure 4:**
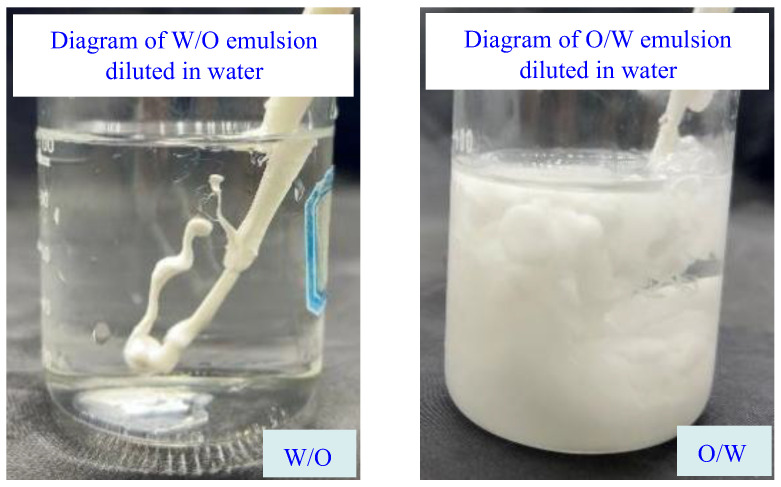
Diagram of dilution of an emulsion (W/O emulsion and O/W emulsion) in water.

It can be seen that the alkali-response phase conversion of the NWX-3 stabilized O/W emulsion is relatively easy, while the NWX-1 stabilized O/W emulsion cannot achieve alkali-response phase transfer, and the emulsion still maintains the O/W emulsion state when the alkali (20 wt.% sodium hydroxide solution) dosage reaches 1.0 vol.%. When the sodium hydroxide concentration reached 1.0 vol.%, the pH of the emulsion system had risen to 11.9. At this time, the concentration of hydroxide ions in the solution is relatively high, and most of the ionic amine groups on the surface of the modified nanocrystalline cellulose have been converted into non-ionic amine groups. Under the pH value, the emulsion remained in the demulsified state and could not be transformed, indicating that the O/W emulsion prepared by the modified nanocrystalline cellulose NWX-1 did not have the function of alkali-response phase transformation. In addition, by testing the dilution of the emulsion formed in water at different stages of phase transfer, it was further confirmed that the NWX-3 stabilized emulsion could achieve a reversible phase (Figure 4), and the modified nanocrystalline cellulose NWX-3 was selected as the emulsifier for the next step of the study of nanoparticle-stabilized reversible drilling fluid.

The demulsification voltage (<100 V) of the W/O emulsion obtained by NWX-3 stabilized emulsion after reversibility was significantly higher than that of HRW-DMOB stabilized emulsion after reversibility (>400 V). The results indicated that the W/O emulsion obtained by NWX-3 stabilized emulsion had a better stability after reversing phase. The demulsification voltage of the initial W/O Emulsion 3 was also significantly higher than that of the surfactant reversible emulsifier system HRW-DMOB, indicating that the reversible emulsion strengthened by NWX-3 was more stable than that of the surfactant before and after the reversible phase.

### 2.3. Mechanism Analysis of Reversible Phase in Emulsions

**Figure 5:**
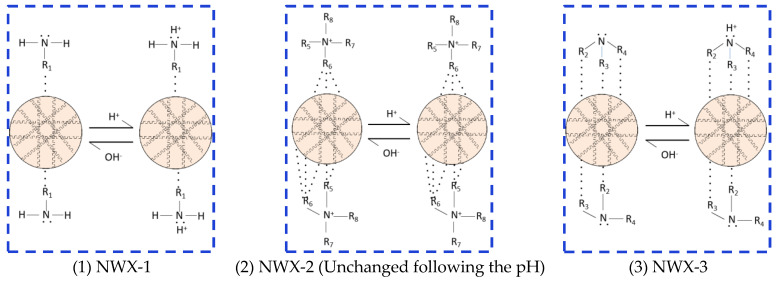
Schematic diagram of the structural change in the modified nanocrystalline cellulose emulsifier during the reversible phase of the emulsion. R1, R2, R3, and R4 are all long-linear alkyl groups. R5, R6, R7, and R8 are all lipophilic groups with multiple branches.

There are lone pairs of electrons in the N atoms of the amine groups of the primary amine surfactant DUW-3 and the tertiary amine surfactant LTS-3, which can be paired with H+ to form a positively charged cationic amine group under acidic conditions, and the hydrophilicity is greatly enhanced. Therefore, the nanocrystalline cellulose NWX-1 modified by the primary amine surfactant DUW-3 and the nanocrystalline cellulose NWX-3 modified by the tertiary amine surfactant LTS-3 can be transformed from lipophilic nanocrystalline cellulose to hydrophilic after the acid response (Figure 5), so that the emulsion can be transformed from W/O to O/W emulsion.

The preferred primary amine surfactant DUW-3 and tertiary amine surfactant LTS-3 are both long single-chain organic amines, and each of their long single chains can form a relatively single link with nanocrystalline cellulose. The primary amine surfactant DUW-3 has only one lipophilic group, while the tertiary amine surfactant LTS-3 has three lipophilic groups. Only one lipophilic group in the primary amine surfactant DUW-3 is adsorbed on the surface of NWX-1, while the lipophilic group of the tertiary amine surfactant LTS-3 is significantly stronger than that of NWX-1 due to the large number of lipophilic groups, and some lipophilic groups are not adsorbed on the surface of NWX-3 (as shown in Figure 5).

There is a hysteresis in the phase inversion of the emulsion [43], and the structure of the O/W emulsion needs to be destroyed before the emulsion can be converted from O/W to W/O [44,45]. Therefore, the emulsion needs to be changed from O/W to W/O compared with the direct preparation of W/O emulsion, which requires a higher lipophilicity of emulsifiers. Although the primary amine surfactant DUW-3 modified nanocrystalline cellulose NWX-1 can prepare the initial W/O emulsion, after its acid response it is converted to an O/W emulsion, and it cannot be effectively converted into a W/O emulsion in the alkali-response stage due to the lack of lipophilicity reflected by NWX-1. However, the nanocrystalline cellulose NWX-3 modified by the tertiary amine surfactant LTS-3 can be effectively converted to the W/O type in the alkali-response stage, so NWX-3 can be used as a reversible emulsifier for modified nanoparticles.

There are no lone pairs of nitrogen atoms on the amine group in the quaternary amine surfactant LHS-5 (Figure 5), and its structure does not change with the addition of acid and alkali. The addition of acid and alkali only affects the diffusion electric double layer of NWX-2, but the diffusion electric double layer has little effect on the hydrophilicity and lipophilicity of the emulsifier, so the properties of NWX-2 are less affected by the addition of acid and alkali. The selected quaternary amine LHS-5 is a multi-branched oil-soluble surfactant, so only some lipophilic groups are adsorbed on the surface of NWX-2 on the surface of nanoparticles, and a large number of lipophilic groups interact with quaternary amine groups to make NWX-2 exhibit strong lipophilicity. Therefore, the nanocrystalline cellulose NWX-2 modified by the quaternary amine surfactant LHS-5 can effectively stabilize the W/O emulsion and does not undergo phase transformation with the addition of acid and alkali.

The reversible emulsion prepared by the surfactant emulsifier was structurally stable, and the reversible emulsifier HRW-DMOB was a composite system of primary amine surfactants. In the acid-response process, the surfactant changes from uncharged R-NH_2_ to positively charged R-NH_3_^+^ (protonation); that is, the surfactant changes from a non-ionic surfactant to a cationic surfactant. In this process, the hydrophilicity of the surfactant is enhanced, and the stabilized emulsion is transformed from a W/O emulsion to an O/W emulsion. In the process of the alkali response, the surfactant changes from positively charged R-NH_3_^+^ to uncharged R-NH_2_ (deprotonation); that is, the surfactant changes from a cationic surfactant to a non-ionic surfactant. In this process, the lipophilicity of the surfactant is enhanced, and the stabilized emulsion is transformed from an O/W emulsion to a W/O emulsion. A Zeta potential and particle size analyzer (Brookhaven company, New York, NY, USA) was used to measure the Zeta potential of CNC samples in a suspension system with a mass fraction of 0.025% at 25 °C. For the modified nanocrystalline cellulose NWX-3 suspension, the zeta potential of the modified nanocrystalline cellulose was further tested under different pH values before and after modification. At pH 10, the zeta potential of the suspension of the unmodified nanocrystalline cellulose was −38.8 mV, and the suspension of the modified nanocrystalline cellulose was 0.3 mV. At pH 4, the zeta potential of the suspension of the unmodified nanocrystalline cellulose was −27.6 mV, and the suspension of the modified nanocrystalline cellulose was 7.9 mV. Through comparison, it can be seen that the modified nanocrystalline cellulose is endowed with pH-corresponding properties, and the properties are significantly different in acidic and alkaline media.

**Figure 6:**
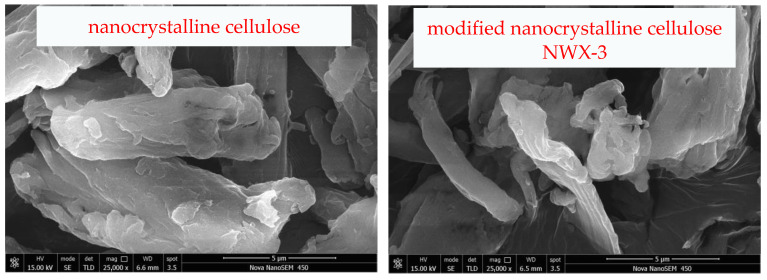
Electron microscopy images of nanocrystalline cellulose and the modified nanocrystalline cellulose NWX-3.

**Figure 7:**
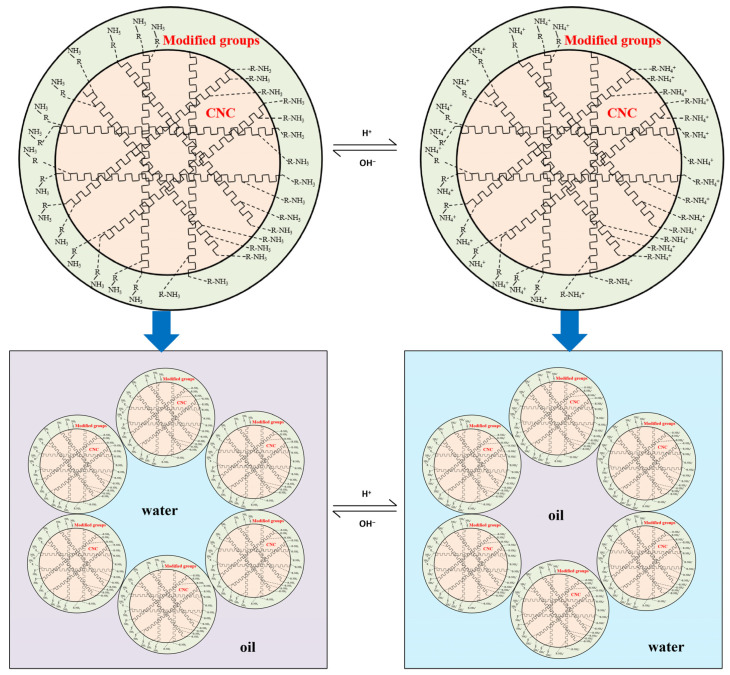
Schematic diagram of the reversible phase process of reversible emulsion prepared by a modified nanocrystalline cellulose emulsifier (NWX-3).

After testing, the nanocrystalline cellulose still retains a small particle size after modification, and the reversible emulsion prepared based on it forms a stable structure at the oil–water interface (Figure 6). The surface charge of the modified nanocrystalline cellulose reversible emulsifier is enhanced during the acid response, resulting in stronger hydrophilicity, and the stabilized emulsion is transformed from W/O to O/W. However, the reduced surface charge of the modified nanocrystalline cellulose reversible emulsifier during the alkali response led to enhanced lipophilicity (Figure 5), and the stabilized emulsion was transformed from O/W to W/O (Figure 7). A change in the properties of the nanocrystalline cellulose (Figure 6) distributed at the oil–water interface (Figure 5) leads to a change in the type of stable emulsion (Figure 7). Comparing the two, the structure formed by the modified nanocrystalline cellulose reversible emulsifier is significantly more stable than that formed by the surfactant reversible emulsifier at the oil–water interface [46], which is consistent with the result that the demulsification voltage of the former is significantly higher than that of the latter.

### 2.4. Preparation of Reversible Emulsion Drilling Fluid

Based on the reversible emulsion prepared by the modified nanocrystalline cellulose emulsifier NWX-3, the types and dosages of wetting agent, leachate reducer, lime, organic soil, and barite were optimized, and the reversible phase performance, stability, temperature resistance, filtration resistance, and density of emulsion drilling fluid were comprehensively evaluated. The formula of reversible emulsified drilling fluid is determined as follows: 5# white oil + 25.0 wt.% calcium chloride aqueous solution + 0.8 wt.% modified nanocrystalline cellulose reversible emulsifier NWX-3 + 1.5 wt.% organic soil + 2.0 wt.% wetting agent LKD + 1.0 wt.% filtration loss reducer DLG + 1.0 wt.% lime + appropriate amount of barite, the oil–water ratio is 60/40, and the density can be adjusted to 1.2 g/cm^3^. The formula of the reversible emulsion drilling fluid system prepared by the surfactant reversible emulsifier HRW-DMOB is 5# white oil + 25.0 wt.% calcium chloride aqueous solution + 1.3 wt.% reversible emulsifier composite system HRW-DMOB + 1.4 wt.% organic soil + 2.5 wt.% wetting agent LND + 1.4 wt.% filtration loss reducer DLK + 1.0 wt.% lime + appropriate amount of barite, the oil–water ratio is 60/40, and the density can be adjusted to 1.2 g/cm^3^. The temperature resistance, reversible phase performance, oil-impregnated drill cuttings treatment performance, and filter cake cleaning performance of the two were evaluated and compared.

### 2.5. Evaluation of Temperature Resistance of Reversible Emulsion Drilling Fluid

The reversible emulsified drilling fluids prepared with NWX-3 as the emulsifier and HRW-DMOB as the emulsion system were subjected to hot rolling experiments (16 h) at different temperatures, and the performance of the drilling fluid before and after aging was compared, and the temperature resistance of the two was evaluated, and the experimental results are shown in Table 1. It can be seen that the properties of the reversible emulsion drilling fluid prepared by HRW-DMOB as the emulsification system change greatly when the temperature is greater than 130 °C, especially the demulsification voltage and high-temperature and high-pressure filtration loss change significantly, indicating that the reversible emulsion drilling fluid can no longer maintain stability at this temperature. The properties of the reversible emulsified drilling fluid prepared with the modified nanocrystalline cellulose NWX-3 as an emulsifier only began to change when the temperature reached 160 °C. Because the structure of the modified nanocrystalline cellulose on the surface of the emulsion is more stable, the temperature resistance of the prepared reversible emulsion drilling fluid is significantly better than that of the former, which is consistent with the experimental results of the demulsification voltage of the reversible emulsion and is also consistent with the structure and mechanism analysis of the reversible emulsion. Huo Jinhua et al. developed a reversible emulsified drilling fluid system sensitive to CTAB concentration, which can be reversed with the increase in CTAB concentration. In terms of temperature resistance, it can effectively withstand 120 °C, which is lower than the reversible emulsified drilling fluid system constructed by the author (effective temperature resistance of 150 °C). In terms of filtration loss, the filtration loss of the system developed by Huo Jinhua et al. was 4.2 mL–5.8 mL, while the filtration loss of the reversible emulsified drilling fluid system developed in this paper was 4.0 mL–6.0 mL, and the filtration loss of the two drilling fluid systems was low [47].

### 2.6. Reversible Phase Inversion Performance Evaluation of Reversible Emulsion Drilling Fluids

According to the above research results, the reversible phase performance of the reversible emulsion drilling fluid prepared with NWX-3 as an emulsifier (hot rolling condition: 150 °C × 16 h) and the reversible phase performance of reversible emulsion drilling fluid prepared with HRW-DMOB as the emulsifier system (hot rolling condition was 120 °C × 16 h) were evaluated. The rheology, filtration, demulsification voltage, and conductivity of the two reversible emulsion drilling fluids were compared under four conditions: before hot rolling, after hot rolling, acid response, and alkali response, and the experimental results are shown in Table 2. It can be seen that the reversible phase performance of the reversible emulsion drilling fluid prepared with NWX-3 as an emulsifier under 180 °C hot rolling conditions and the reversible phase performance of the reversible emulsion drilling fluid prepared with HRW-DMOB as an emulsifier system under 120 °C hot rolling conditions are good, and there is no significant change in the properties of the two before and after phase inversion.

### 2.7. Oily Drill Cuttings Treatment Performance of Reversible Emulsion Drilling Fluid

With the increasing use of oil-based drilling fluids, the treatment of oil-bearing drill cuttings has gradually attracted people’s attention. At present, the main methods used are the biodegradation method, solvent extraction method, and high-temperature pyrolysis method, which have the disadvantages of high cost and complex operation. This problem can be solved by using a reversible emulsion drilling fluid, which can realize the oil-based drilling fluid in the drilling stage and has the advantages of oil-based drilling fluid. Moreover, acid is used in the treatment of oil-bearing drill cuttings, so that the surface of oil-bearing drill cuttings is reversed from lipophilic to hydrophilic, so as to solve the treatment problem of oil-bearing drill cuttings. The experimental results are shown in Table 3 for comparing the oil-bearing drill cuttings treatment performance of conventional oil-based drilling fluid and NWX-3 stabilized reversible emulsion drilling fluid. It can be seen that the oil-bearing drill cuttings produced by the stable reversible emulsified drilling fluid of NWX-3 can be separated from the oil phase after acid treatment, and the separation time of the two cuttings is about 5 h.

### 2.8. Filter Cake Treating Performance of the Drilling Fluid

**Figure 8:**
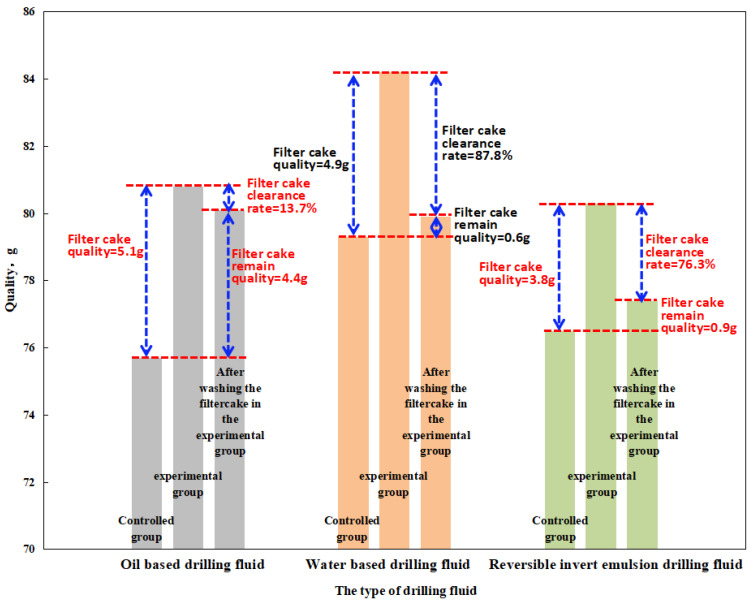
The filter cake treating performance of the drilling fluid.

Oil-based filter cake will be generated between the wellbore wall and casing after the operation of oil-based drilling fluid, which will affect the next cementing operation. In order to remove damage from oil-based filter cake, it is necessary to remove the cake with a scavenger prior to cementing when using oil-based drilling fluids. At present, the main component of the filter cake scavenger is a surfactant, and the use of the scavenger will not only increase the cost, but also cause environmental pollution. The use of a reversible emulsion drilling fluid system can avoid the problem of oil-based filter cake cleaning on the basis of ensuring the drilling effect of oil-based drilling fluid. The reversible emulsion drilling fluid system can be used as an oil-based drilling fluid during the drilling phase. However, in the completion stage, the oil-based mud cake can be removed by injecting acid, and the drilling and completion operation can be completed using only the conventional water-based drilling fluid. The cleaning effects of the reversible emulsified drilling fluid RINW-7 constructed by modified nanocrystalline cellulose NWX-3, the oil-based drilling fluid HHD-O3, and the water-based drilling fluid HHD-9 from Shengli Oilfield were compared, and the experimental results are shown in Figure 8.

A comparison group and an experimental group were set up, wherein (1) represents the comparison group: weighing flask + treatment agent + filter paper (drilling fluid infiltration); (2) represents the experimental group: weighing flask + treatment agent + filter paper (with filter cake after filtration); and (3) represents the experimental group rinsed with acid solution (mass fraction of 5.0 wt.%). The oil-based drilling fluid HHD-O3 in Shengli Oilfield, HHD-9 in Shengli Oilfield, and RINW-7 based on the modified nanocrystalline cellulose NWX-3 were used as drilling fluids.

Compared with the cleaning effect of the filter cake obtained under high temperature and high pressure (Figure 8), it can be seen that the reversible emulsion drilling fluid RINW-7 constructed based on the nanocrystalline cellulose NWX-3 has a significantly better cleaning effect than the oil-based drilling fluid HHD-O3 acid in Shengli Oilfield, which is close to the cleaning effect of the water-based drilling fluid HHD-9.

### 2.9. Reservoir Protection Performance of Reversible Emulsion Drilling Fluid

**Figure 9:**
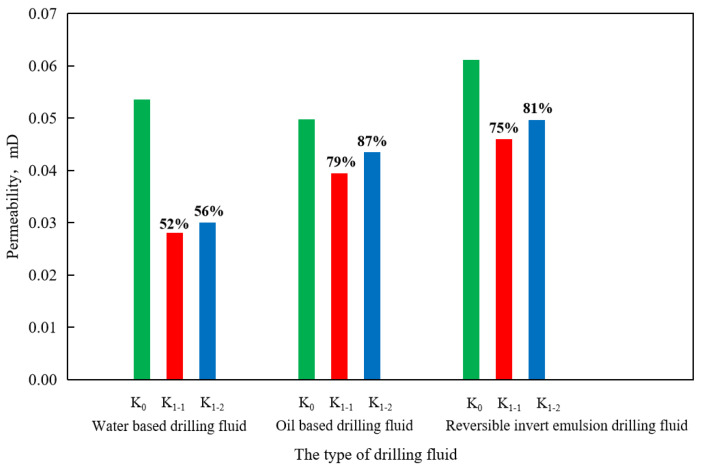
Damage of different types of drilling fluids to reservoir gas permeability.

Based on the above-mentioned design of the reservoir damage evaluation process, the reservoir permeability damage of oil-based drilling fluid, water-based drilling fluid, and reversible emulsion drilling fluid was compared by the physical simulation method (Figure 9). It was found that the damage to the permeability of the core gas is as follows: water-based drilling fluid > reversible emulsion drilling fluid > oil-based drilling fluid, and the damage degree of reversible emulsion drilling fluid to core gas permeability was not much different from that of the oil-based drilling fluid; that is, the reverse emulsion drilling fluid had a better protective effect on the reservoir permeability than the water-based drilling fluid. Compared with the oil-based drilling fluid, the reversible emulsified drilling fluid RINW-7 constructed by combining the above-mentioned modified nanocrystalline cellulose NWX-3 had a better protective effect on the permeability of the reservoir.

**Figure 10:**
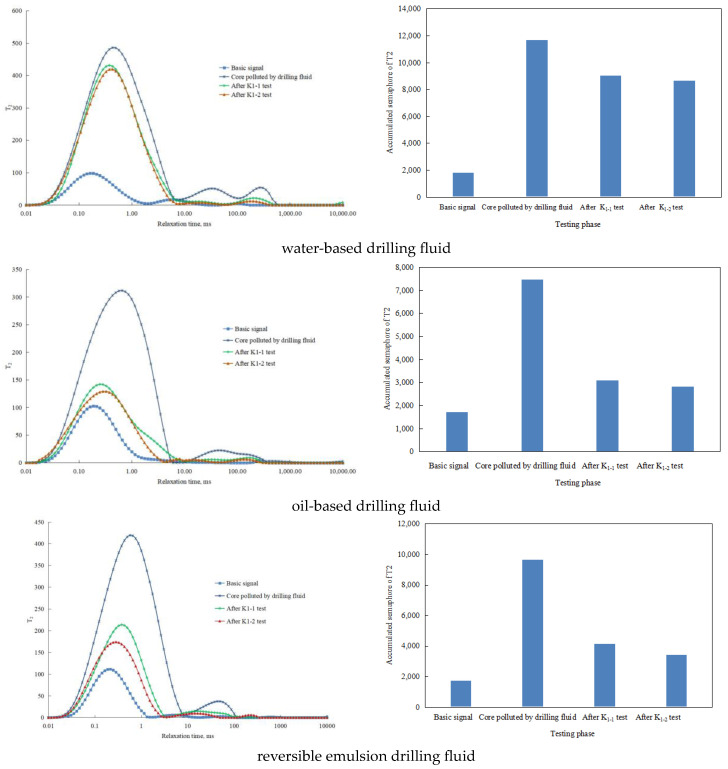
NMR test results and cumulative signal changes in different types of drilling fluids at different stages of reservoir damage.

Nuclear magnetic resonance technology is a high-tech technology applied in the field of petroleum exploration and development in recent years, which has the characteristics of rapid, non-destructive, multi-parameter, and multi-dimensional measurement and is an important means for accurate characterization of rock pore structure and fluid saturation. At present, researchers have carried out relevant research and application of NMR technology in pore throat characterization and reservoir evaluation in shale reservoirs and have found that when the fluid is in a small space, its NMR relaxation time is significantly reduced compared with the free state. Therefore, the author analyzed the intrusion of different types of drilling fluids into the core and the retention of drilling fluids in the core after gas flooding by comparing the equilibrium permeability of K1-1 before core damage, after drilling fluid intrusion and damage, and the equilibrium permeability of K1-2 after 60 min of forward displacement to reach the stable pressure and flow rate (Figure 10).

By comparing the NMR states of different types of drilling fluids (oil-based drilling fluids, water-based drilling fluids, and reversible emulsion drilling fluids) as working fluids, the NMR states corresponding to different stages of the core (before core injury, after drilling fluid intrusion injury, equilibrium gas permeability K1-1 test, and equilibrium permeability K1-2 test after positive displacement after 60 min of stable pressure and flow) (Figure 10). It is clear that the order of fluid intrusion and fluid retention after gas flooding is as follows: water-based drilling fluid > reversible emulsion drilling fluid > oil-based drilling fluid. Considering that the more intrusive fluid is retained after air flooding, the more serious the damage is, the results of this NMR test are consistent with the permeability damage ranking of the aforementioned tests. Therefore, the main cause of core permeability damage is the damage caused by the retention of intrusive fluid in the core.

## 3. Materials and Methods

### 3.1. Materials

The primary amine surfactant DUW-3 (Dodecylamine), quaternary amine surfactant LHS-5 (Hexadecyltrimethylammonium chloride), tertiary amine surfactant LTS-3 (Lauramide propyl dimethyl tertiary amine), hydrochloric acid, sodium hydroxide, calcium chloride, calcium oxide, and nanocrystalline cellulose CNC-4 were purchased from Sinophosphate Chemical Reagent Co., Ltd. (Beijing, China). The reversible emulsifier system HRW-DMOB (Laboratory construction of primary amine surfactant complex system, HRW is Octadecylamine, DMOB is N,N-dimethyl-N’-oleoyl-1,4-butanediamine) was self-made in the laboratory. The 5# White oil was purchased from the Scaran Group in France. The organic soil, Wetting agent LKD, Fluid loss reducer DLG, and Barite were offered by Shengli Oilfield Drilling Engineering Technology Co., Ltd. (Dongying, China). The core of the shale gas reservoir was provided by the Shengli Oilfield Company, Sinopec (Dongying, China).

### 3.2. Preparation of Modified Nanocrystalline Cellulose

**Figure 11:**
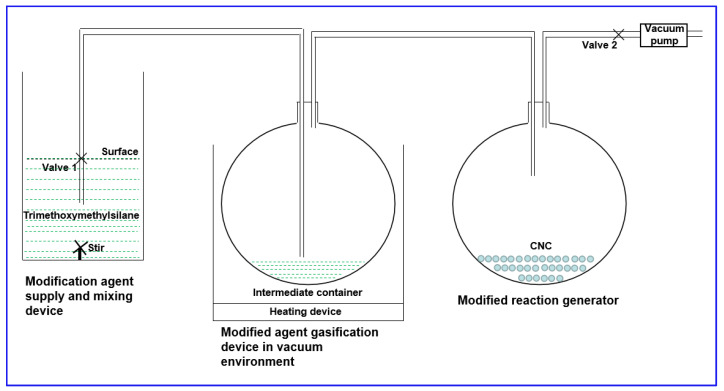
Experimental process for the preparation of lipophilic nanocrystalline cellulose.

Nanocrystalline cellulose with a strong oleophilic surface was prepared based on the designed experimental flow (Figure 11): Close Valve 1, open Valve 2, use the vacuum pump to vacuum for 2–3 h; close Valve 2, open Valve 1, and introduce an appropriate amount (<1/4 volume of the flask) of methyltrimethoxysilane into Flask 1. The oil bath was used to heat Flask 1, and the oil bath was kept at 90 °C for 10 h, so that the nanocrystalline cellulose was fully modified (m _organosilanizing agent of flask 1_:m _nanocrystalline cellulose_ = 3:1), and nanocrystalline cellulose with a strong oleophilicity was obtained, which was named nanoparticle ①.

In total, 3 parts of nanoparticles ① were added into 200 mL of anhydrous ethanol, stirred at a uniform speed of 300 r/min for 20 min, and dispersed by ultrasonic at room temperature for 30 min to obtain a fully dispersed suspension system. The suspension system was added into a 250 mL three-mouth flask, 2 parts of organic amine surfactant were slowly added to it, and stirring was continued for 5 h. After the product was cleaned with anhydrous ethanol 3 times, pH-responsive nanocrystalline cellulose was obtained by vacuum drying at 50 °C for 12 h.

### 3.3. Preparation of Reversible Emulsion Drilling Fluid

An appropriate amount of emulsifier was added into the white oil and stirred at 12,000 r/min for 5 min (GJSS-B12K inverter high speed mixer, Jiaonan Analytical Instrument Factory, Qingdao, China). An appropriate amount of water phase (25.0 wt.% calcium chloride aqueous solution) was added and stirred at the same speed for 40 min. Then, an appropriate amount of organic soil, fluid loss reducer, calcium oxide, wetting agent, and barite were added in turn and stirred for 10 min to prepare the reversible emulsion drilling fluid.

### 3.4. Performance Test of Reversible Emulsion Drilling Fluid

#### 3.4.1. Test of Reversible Phase Performance for Reversible Emulsion Drilling Fluid

A high-temperature roller heating furnace (Qingdao Haitongda Special Instrument Co., Ltd., Qingdao, China) was used for the hot rolling of the reversible emulsion drilling fluid. An appropriate amount of hydrochloric acid (20.0 wt.%) was added to the reversible emulsion drilling fluid after hot rolling and stirred at 12,000 r/min at high speed for 5 min to test the emulsion type. An appropriate amount of sodium hydroxide solution (20.0 wt.%) was added to the reversible emulsion drilling fluid obtained above and stirred at 12,000 r/min for 5 min to test the emulsion type.

#### 3.4.2. Routine Performance Test of Reversible Emulsion Drilling Fluid

A GGS42-1 filtration instrument (Qingdao Haitongda special Instrument Factory, Qingdao, China) was used to determine the high temperature and pressure filtration loss of the reversible emulsion drilling fluid. A ZNS-1 mud water loss measuring instrument (Jiaonan Analytical Instrument Factory, Qingdao, China) was used to measure the normal temperature and atmospheric pressure filtration loss of the reversible emulsion drilling fluid. The conductivity of the reversible emulsion drilling fluid was tested with a DDS-307 conductivity meter (Shanghai YIZhuang Scientific Instrument Co., Ltd., Shanghai, China). The rheological properties of the reversible emulsion drilling fluid were measured by a ZNN-D6 six-speed rotating viscometer (Qingdao Haitongda Special Instrument Co., Ltd., Qingdao, China): AV = θ_600_/2, PV = θ_600_ − θ_300_, YP = 0.511(θ_300_ − PV); The demulsification voltage of the reversible emulsion drilling fluid was measured by a DWY-2 electrical stability tester (Qingdao Haitongda Special Instrument Factory, Qingdao, China).

#### 3.4.3. Evaluation of Oil-Bearing Cuttings Treatment Performance of Reversible Emulsion Drilling Fluid

A certain amount of drilling cuttings was added to the reversible emulsion drilling fluid and heated for 2 h at 150 °C, then the drilling cuttings were separated and soaked in 20.0 wt.% hydrochloric acid solution for 2 h, and the immersed drilling cuttings were added to clean water to record the separation time of the cuttings.

#### 3.4.4. Evaluation of Filter Cake Treatment Performance of Reversible Emulsion Drilling Fluid

The reversible emulsion drilling fluid RINW-7 (150 °C, 3.5 MPa) constructed by nanocrystal cellulose NWX-3 was used in filter cake removal by high temperature and pressure filtration with the oil-based drilling fluid HD-O3 (120 °C, 3.5 MPa) and water-based drilling fluid HHD-9 (120 °C, 3.5 MPa) of Shengli Oilfield. The filter cake was soaked in 20.0 wt.% hydrochloric acid for 30 min, and kept gently shaken and agitated during the soaking process, and the filter cake would gradually disengage. The comparison group and the experimental group were set, among which ① was the comparison group: weighing bottle + treatment agent + filter paper (drilling fluid infiltration); ② was the experimental group: weighing bottle + treatment agent + filter paper (with filter cake after filtration); and ③ represented the experimental group washed with acid solution (mass fraction 5.0 wt.%). The quality change in different groups was weighed and the pickling effect of the filter cake produced by different types of drilling fluid was analyzed and compared.

#### 3.4.5. Evaluation of Reservoir Protection Effect of Reversible Emulsion Drilling Fluid

(1) Determine the gas permeability K0 of the core before damage

**Figure 12:**
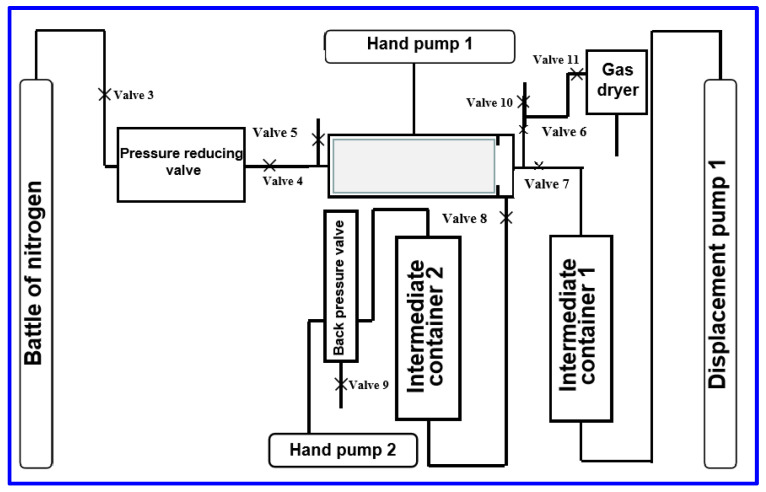
Flow chart of drilling fluid damage evaluation experiment.

**Figure 13:**
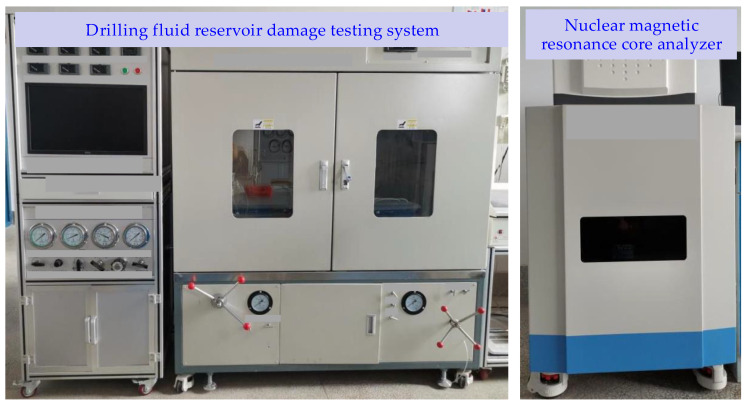
Drilling fluid reservoir damage testing and analysis system.

The core was tested by nuclear magnetic resonance (NMR core Analyzer, Suzhou Niumai Analytical Instruments Co., Ltd., Suzhou, China) to obtain the basic nuclear magnetic signal of the core and set as a comparison with subsequent test results. Using the drilling fluid damage assessment experiment system (Figure 12 and Figure 13), the pre-prepared core was loaded into the rubber sleeve of the core holder. The process was as follows: Open Valve 3, Valve 4, Valve 6, and Valve 11, close Valve 5, Valve 7, Valve 8, and Valve 10 to adjust the ring pressure to 2–3 Mpa, and use a nitrogen cylinder to pressure the core through the pressure reducing valve to keep the pressure constant displacement to a stable flow and stable pressure, then measure the gas permeability of the core before damage. The equilibrium gas permeability K_0_ of the core before damage is calculated by Darcy’s formula. Fill the water phase under the piston and the evaluation liquid (drilling fluid for pollution) above the piston in Intermediate Container 1. Fill the water phase under the piston in Intermediate Container 2 and add the water phase above the piston.

(2) Drain

Open Valve 7 and empty Valve 6, Valve 5, close Valve 8, Valve 4, and Valve 3 to adjust the ring pressure to 2–3 Mpa (pay attention to observe whether the pressure is stable after the confining pressure is added to the required value: If it is not stable, it indicates that there is confining pressure leakage, check whether the rubber sleeve is damaged or the core diameter is too small; if the rubber sleeve is damaged, replace the rubber sleeve; if the core is too small or improperly installed, a layer of Teflon film can be wrapped around the core and re-installed until the confining pressure is stable). Displacement Pump 2 is used to slowly pressurize Intermediate Container 4, and the evaluation hydraulic pressure in the intermediate vessel is put into the experimental liquid chamber at the entrance end of the fluid loss test core holder. When the evaluation liquid flows out of the vent of Valve 6, close Displacement Pump 2 and close the vent of Valve 6.

(3) Adjust the pressure

Adjust the pressure of Hand Pump 2 and make the pressure of the return pressure valve equal to the inlet pressure. Adjust the thermostat temperature. The adjustment of the confining pressure is carried out in two steps. Firstly, the confining pressure of the core is increased to 3 MPa, the pressure in the experimental liquid chamber is increased to 2.5 MPa (or lower than the required experimental pressure of 0.5 MPa) with Displacement Pump 1, and then the confining pressure is increased to 4.5–5 MPa (or higher than the experimental pressure of 1.5–2 MPa). Then, use Displacement Pump 1 to increase the pressure in the experimental liquid chamber to 3.5 MPa (or the required experimental pressure).

(4) Dynamic fluid loss injury

When the temperature and pressure reach the experimental value, open Valve 7, Valve 8, Valve 9, and Valve 5, set Displacement Pump 1 to a constant flow rate mode, and start the dynamic filtration test. During the experiment, the volume of filtrate was recorded at different times. The dynamic filtration test was completed after 125 min.

(5) End dynamic filtration test

After the end of the dynamic filtration test, close the filtrate outlet valve immediately, stop the heating, and carry out cooling, and carry out slow agitation. When the temperature drops to 50 °C, the pressure can be relieved, the rotation can be stopped, the experimental liquid can be released, the core can be removed, and the thickness of the mud cake can be measured. Clean the fluid loss meter immediately and wipe it dry.

(6) The gas permeability K_1_ of the core is measured after the drilling fluid damages the core

Close all valves, open Valve 3, Valve 4, Valve 5, Valve 6, and Valve 11, and let the exhaust inlet filtrate with nitrogen. The same flow rate as that used to determine the core K_0_ is adopted. When the pressure flow rate is initially stabilized, the equilibrium gas permeability K_1-1_ of the damaged core is calculated by Darcy’s formula, and the nitrogen displacement is continued forward for 60 min. After the stable pressure and flow rate are reached, the gas permeability of the core after the drilling fluid damages the core is measured. The equilibrium gas permeability K_1-2_ of the damaged core is calculated by Darcy’s formula. At the same time, observe and record the highest flowback pressure difference during displacement.

## 4. Conclusions

The reversible emulsion prepared by the modified nanocrystalline cellulose NWX-3 not only has a small emulsifier dosage (0.8 wt.%), strong stability (1000 V), and good temperature resistance (150 °C), but also maintains a good reversible phase, filter cake removal, and oil-impregnated drill cuttings treatment effects. It provides a feasible scheme for the preparation of reversible emulsion drilling fluid. In addition, the damage of the reservoir permeability of different types of drilling fluids was compared by physical simulation, and the damage order of core gas permeability was clarified: water-based drilling fluid > reversible emulsion drilling fluid > oil-based drilling fluid. Further comparing the NMR states of the corresponding cores when different types of drilling fluids are used as working fluids, it is clear that the order of fluid intrusion and fluid retention after gas flooding is as follows: water-based drilling fluid > reversible emulsion drilling fluid > oil-based drilling fluid, and the main reason for core permeability damage is the retention of intrusive fluid in the core.

## Figures and Tables

**Table 1:** Characterization of temperature resistance of reversible emulsion drilling fluids.

	HRW-DMOB Emulsification System				Modified Nanocrystalline Cellulose NWX-3 Emulsion System			
Temperature/ °C	PV/ mPa.s	YP/ Pa	ES/ V	FL_HTHP_/ mL	PV/ mPa.s	YP/ Pa	ES/ V	FL_HTHP_/ mL
Before aging	51	14	650	–	52	11	1147	–
100	53	13	630	4	53	13	1185	5
120	54	14	615	4	51	13	1203	4
130	62	16	48	31	54	12	1164	6
140	65	17	17	34	53	11	1121	5
150	69	20	0	41	52	13	1106	5
160	–	–	–	–	65	18	68	32
180	–	–	–	–	–	–	–	–

Note: The density of the system: 1.20 g/cm^3^; FLHTHP: 3.5 MPa.

**Table 2:** The reversible phase inversion performance of the reversible invert emulsion drilling fluid.

	Emulsifier HRW-DMOB Stabilized Reversible Emulsion Drilling Fluid (Hot Rolling Conditions: 120 °C × 16 h)				Emulsifier NWX-3 Stabilized Reversible Emulsion Drilling Fluid (Hot Rolling Conditions: 150 °C × 16 h)			
	Before Hot Rolling	After Hot Rolling	Acid Response	Alkali Response	Before Hot Rolling	After Hot Rolling	Acid Response	Alkali Response
Type	W/O	W/O	O/W	W/O	W/O	W/O	O/W	W/O
PV/mPa.s	50	51	20	49	52	53	15	50
YP/Pa	12	13	5	12	11	11	4	12
API/mL	0.1	0.2	1.1	0.7	0.2	0.1	1.5	0.5
FL_HTHP_/mL	–	7	12	7	–	8	13	9
Demulsification voltage/V	635	619	0	608	1147	1079	0	1022
electrical conductivity /S.m^−1^	0	0	7500	0	0	0	9337	0
pH	8	8	6.5	8	8	8	6.6	8

**Table 3:** Oily drill cuttings treatment performance of reversible emulsion drilling fluid.

Condition	Time-Consuming for Complete Dispersion of Oil-Bearing Drill Cuttings	
	Conventional Oil-Based Drilling Fluids	NWX-3 Stabilized Reversible Emulsified Drilling Fluid
untreated	nondispersed	nondispersed
acid treatment	nondispersed	5 h completely dispersed

## Data Availability

The data presented in this study are available wholly within the manuscript.
